# Acute Anxiolytic Effects of *Salvia Heldreichiana* Essential Oil in Rats: Reduction in Serum Cortisol

**DOI:** 10.1002/brb3.70701

**Published:** 2025-09-08

**Authors:** Ahmet Onur Daştan, Aslı Şan Dağlı Gül, Gülderen Yılmaz, Okan Arıhan, Ayşen Erdem

**Affiliations:** ^1^ Faculty of Medicine, Department of Physiology Hacettepe University Ankara Türkiye; ^2^ Faculty of Pharmacy, Department of Pharmaceutical Botany Ankara University Ankara Türkiye

**Keywords:** anxiolytic, behavior, cortisol, hippocampus, salvia heldreichiana

## Abstract

**Purpose:**

The rapid onset of anxiolytic drugs without cognitive or motor impairments remains an unmet need. This study evaluated the acute anxiolytic effects of *Salvia heldreichiana* essential oil in rats, measuring anxiety‐related behaviors, hippocampal levels of serotonin, noradrenaline, gamma‐aminobutyric acid GABA, and serum cortisol.

**Method:**

Forty‐eight male Wistar albino rats were divided into two experiments. Experiment 1 (*n* = 24) tested a single dose (200 mg/kg, oral gavage) of *Salvia heldreichiana* essential oil, while Experiment 2 (*n* = 24) evaluated repeated doses (200 mg/kg/day for five days, oral gavage). Anxiety‐like behavior was assessed using the open field and elevated plus maze (EPM) tests. Sedation and motor coordination were measured via the rotarod test. Neurochemical analysis included hippocampal noradrenaline, serotonin, GABA levels, and serum cortisol. Potential toxicity was monitored through ALT, AST, creatinine, and BUN levels.

**Findings:**

*Salvia heldreichiana* essential oil significantly reduced serum cortisol levels (*p* < 0.05) and showed a trend toward increased hippocampal serotonin levels, similar to diazepam. No sedative or motor impairing effects were observed, and no hepatotoxic or nephrotoxic effects were observed at the administered dose.

**Conclusion:**

*Salvia heldreichiana* essential oil showed promise as a rapid‐onset anxiolytic, as it reduced cortisol levels and may increase serotonin, all without causing motor impairments. Further studies with conditioned anxiety models are required to confirm these effects and elucidate underlying mechanisms.

**Significance Statement:**

This study highlighted the potential of *Salvia heldreichiana* essential oil as a rapid‐onset anxiolytic with minimal side effects. The oil significantly reduced serum cortisol levels, a key biomarker of stress, and showed a trend toward increased serotonin levels in the hippocampus, contributing to its anxiolytic effects, paralleling mechanisms observed in conventional anxiolytic medications. Unlike many existing treatments, *Salvia heldreichiana* did not induce sedation or impair motor coordination, highlighting its promise as a safer alternative for anxiety management. These findings suggested the essential oil could address the unmet need for fast‐acting anxiety treatments without cognitive or physical drawbacks, making it relevant for both clinical and general use.

## Graphical abstract

Administration of a single and repeated dose (five days) of 200 mg/kg *Salvia heldreichiana* essential oil resulted in a reduction of serum cortisol levels in rats.

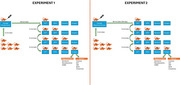

## 1 Introduction

Anxiety disorders are among the most common psychological disorders worldwide and in all age groups. They constitute an important socioeconomic burden in society (Merikangas and Eun 2017; Stein and Sareen 2019; Wittchen and Baum 2020, Boland and Verduin 2022; Ravindran and Stein 2017). Psychological treatment methods, pharmacologic agents, or combinations of both are used in the treatment of anxiety disorders (Stein and Sareen 2019; Ravindran and Stein 2017). Selective serotonin reuptake inhibitors (SSRIs) and serotonin noradrenaline reuptake inhibitors (SNRIs) are used as first‐line drugs among pharmacologic treatments (Boland and Verduin 2022; Ravindran and Stein 2017). Response to treatment with these drugs is observed after 2–4 weeks at the earliest (Ravindran and Stein 2017; Glass et al. 2018). They cannot be used as acute anxiolytics because of the late onset of effects (Sartori and Singewald 2019). In addition, SSRIs may worsen anxiety symptoms at the beginning of treatment (Glass et al. 2018; Sartori and Singewald 2019). Another drug group used in anxiety disorders is benzodiazepines. They may be preferred in the acute period because of their rapid onset of action (Ravindran and Stein 2017; Glass et al. 2018). However, it is emphasized that patients should be warned about not driving and using dangerous equipment during their use because of side effects including, dizziness, sedation, drowsiness, and motor coordination disorder (Ravindran and Stein 2017; Choy 2023).

Various neuroanatomical structures contribute to the development of anxiety. The hippocampus is important in environmental contextual analysis in animals. It plays a role in associating threatening stimuli with the environment (Gee and Casey 2017). In humans, the hippocampus is also involved in contextual conditioning (Gee and Casey 2017) and discrete cue conditioning (analyzing cues representing danger and safety) (Leicht and Mulert 2020; Lissek et al. 2014). Key neurochemicals linked to anxiety include noradrenaline, GABA, serotonin, and cortisol, all of which are associated with the hippocampus (Glass et al. 2018, Gee and Casey 2017; Feder et al. 2017).

The genus *Salvia* is the largest member of the Lamiaceae family and approximately 980 species belonging to this genus have been described (Hu et al. 2018). Among these species, *Salvia miltiorrhiza* (Liu et al. 2015; Lobina et al. 2018; Lin et al. 2021), *Salvia officinalis* (Choukairi et al. 2019; Zeidabadi et al. 2020), *Salvia elegans* (Herrera‐Ruiz et al. 2006; Mora et al. 2006), *Salvia reuterana* (Rabbani et al. 2005) and *Salvia limbata* (Jahani et al. 2022) have shown anxiolytic‐like effects. *Salvia heldreichiana* Boiss. ex Bentham is an endemic species of Türkiye (Basalma et al. 2007; Akin et al. 2010). Mostly α‐pinene (Basalma et al. 2007), linalool (Akin et al. 2010), and β‐pinene (Erdoğan 2014) were detected in the essential oil of this species. In the literature, there are studies indicating that these components show antidepressant and anxiolytic‐like effects (Linck et al. 2010, Kasuya et al. 2015; Guzmán‐Gutiérrez et al. 2015; Weston‐Green et al. 2021). Apart from these components, *Salvia heldreichiana* essential oil contains high levels of spatulenol, caryophyllene oxide, 1,8‐cineole, borneol, and camphor (Basalma et al. 2007; Akin et al. 2010; Erdoğan 2014). In terms of its content, it is similar to *Salvia officinalis* (Raal et al. 2007), *Salvia elegans* (Ali et al. 2015), and *Salvia limbata* (Rajabi et al. 2014) species, which have been shown to reduce anxiety‐like behaviors. The observation of anxiolytic‐like effects in other species of the same genus and the similar chemical composition with these species suggest that *Salvia heldreichiana* may also exhibit anxiolytic‐like properties. However, differences in phytochemical composition or concentration between species may lead to varying anxiolytic effects or reduced a side effect profile.

Current treatment options for anxiety disorders present two challenges: patients must either endure symptoms for 2–4 weeks until medication takes effect, or choose treatments that may impair cognitive and motor function, potentially disrupting work or school life. Requesting that an individual with severe anxiety, who is struggling to manage their symptoms, hold on for a few more weeks may be challenging. On the other hand, interrupting the patient's school or work life, or even labeling them due to their psychiatric illness may have negative career implications. Therefore, there is a need for new anxiolytic drugs that have a rapid onset of action but do not cause cognitive and motor dysfunction. Many plant‐derived extracts or active ingredients are being studied in the hope of answering this need. In this study, the acute anxiolytic effect of *Salvia heldreichiana* essential oil was assessed on rats.

## 2 Materials and Methods

### 2.1 Experimental Animals and Housing Conditions

48 male Wistar albino rats (6–7 weeks old, 200–300 grams) were used. They were housed in a 22 ± 2°C room with 40–45% humidity and a 12‐hour light/dark cycle, in Plexiglas cages (35 × 56 × 19 cm) with four rats per cage. Aspen substrate (TAPVEI, Estonia) was used on the cage floor. Food and water were provided ad libitum. Ethics approval was obtained from the Local Ethics Committee (2021/05‐04), and all protocols followed animal welfare standards.

### 2.2 Obtaining Salvia Heldreichiana Essential Oil

*Salvia heldreichiana* was collected in July 2021 from the slopes of Yeşildere Village, Karaman Province, Türkiye, with permission from the Ministry of Agriculture and Forestry. The plant was identified by pharmaceutical botanist Gülderen Yılmaz and deposited at the Ankara University Faculty of Pharmacy Herbarium under accession number AEF 30892. The above‐ground parts of *Salvia heldreichiana* were shade‐dried, pulverized, and distilled using a Clevenger‐type apparatus for three hours. From 150 g of dried plant material, approximately 0.5 mL of essential oil was obtained, corresponding to a 0.33% (v/w) yield per distillation. To obtain the total amount required for the experiments, the distillation was repeated on consecutive days. The collected essential oil was stored at +4°C in sealed glass containers until use.

Since no prior study involving *Salvia heldreichiana* essential oil was found in the literature, the dose of 200 mg/kg was determined based on a previous study using *Salvia miltiorrhiza* essential oil (Liu et al. 2015) and our preliminary pilot experiment (Dastan et al. 2022) evaluating behavioral responses. Although essential oils may possess irritant properties, the oil was substantially diluted prior to administration, and all animals were fed ad libitum, minimizing gastrointestinal sensitivity. No signs of pain, agitation, or adverse reactions were observed following oral gavage. Furthermore, during subsequent surgical procedures, no macroscopic signs of gastrointestinal irritation, ulceration, or inflammation were observed, suggesting that the administered dose and vehicle were well tolerated.

### 2.3 Pharmaceuticals and Chemicals

Diazepam (Diazem 5 mg capsule, Deva Holding, Türkiye), with anxiolytic and sedative effects, was administered to the diazepam group. Tween 20 (Fisher BioReagents, LOT 172023, USA) was given to all groups as the vehicle.

### 2.4 Behavioral Experiments

#### 2.4.1 Open Field Test (OFT)

A 100 × 100 × 50 cm apparatus (Janthakhin et al. 2017) was used in the experiment, with the base divided into 25 squares measuring 20 × 20 cm (Duszczyk et al. 2015) and illuminated at approximately 400 lux (Riebe and Wotjak 2012). The inner region of nine squares was defined as the center and the edges of 16 squares as the outer region. Rats were placed in the center of the field. An observer, hidden from view, recorded the movements of the rats for five minutes (Riebe and Wotjak 2012) with an overhead camera. After each test, the apparatus was cleaned with 70% ethanol (Duszczyk et al. 2015). The recorded videos were analyzed for the time spent in the center, number of entries into the center, duration of thigmotaxis, number of thigmotaxis, number of rearing, number of grooming, duration of grooming, and number of defecations. Entry into the center was defined as the condition in which all four paws were within the center, while thigmotaxis was defined as the condition in which all four paws were in the outer region (Riebe and Wotjak 2012).

#### 2.4.2 EPM Test

A plexiglass apparatus with four arms measuring 50 × 50 × 10 cm and a 10 × 10 cm intersection area was used. Three sides of the closed arms were enclosed with 40 cm high plexiglass walls, and the apparatus was elevated 70 cm from the ground on four wooden legs (Riebe and Wotjak 2012). Rats were placed at the intersection of the arms, facing the open arm opposite the experimenter (Ari et al. 2019). An observer, hidden from view, recorded the movements of the rats for five minutes (Riebe and Wotjak 2012) using an overhead camera. After each test, the apparatus was cleaned with 70% ethanol. The recorded videos were analyzed for the number of entries into closed and open arms, percentage of entries into open arms (PEOA)*, time spent in closed arms and open arms, and percentage of time spent in open arms (PTSOA)**. Entry into an arm was defined as the rat having all four paws within that arm. Rats that fell out of the maze were quickly placed back into the open arm, but their data were not included in the analysis (Walf and Frye 2007).

$$*\mathrm{PEOA}=\frac{\mathrm{Number}\;\mathrm{of}\;\mathrm{entries}\;\mathrm{into}\;\mathrm{open}\;\mathrm{arms}}{\mathrm{Number}\;\mathrm{of}\;\mathrm{entries}\;\mathrm{into}\;\mathrm{open}\;\mathrm{arms}+\mathrm{Number}\;\mathrm{of}\;\mathrm{entries}\;\mathrm{into}\;\mathrm{closed}\;\mathrm{arms}}\times 100$$

$$**\mathrm{PTSOA}=\frac{\mathrm{Time}\;\mathrm{spent}\;\mathrm{in}\;\mathrm{open}\;\mathrm{arms}}{\mathrm{Time}\;\mathrm{spent}\;\mathrm{in}\;\mathrm{open}\;\mathrm{arms}+\mathrm{Time}\;\mathrm{spent}\;\mathrm{in}\;\mathrm{closed}\;\mathrm{arms}}\times 100$$

#### 2.4.3 Rotarod Test

An 8 cm diameter cylinder rod, capable of rotating 16 times per minute, was used. Rats were allowed a one‐minute rest before testing. They were then held by their tails and placed in one of four separate 10 cm‐wide compartments on the rod, facing the opposite direction of rotation. Once the rats adapted to the rod's movement, their tails were released, and a stopwatch was started. Each rat remained on the rod until it fell or completed 60 s. The rat that fell or completed 60 s was returned to its cage for one minute of rest. Each rat underwent a total of five trials with one‐minute intervals, with the first two serving as practice and the last three as test phases. A rat that completed the test phases without falling received the maximum score of 180 points (3 × 60 s) (Ferrante et al. 2002). Reduced time on the apparatus indicated impaired motor coordination and sedation. The test was recorded using a cell phone camera from the opposite side.

### 2.5 Experimental Groups and Protocols

The study consisted of Experiment 1, which examined the effects of a single dose of *Salvia heldreichiana* essential oil, and Experiment 2, which assessed the effects of a repeated dose (five days).

#### 2.5.1 Experiment 1 (Single Dose)

In Experiment 1, 24 rats were divided into three groups of eight with similar weight: control, diazepam (1.5 mg/kg diazepam), and Salvia (200 mg/kg *Salvia heldreichiana* essential oil). All substances, including a 0.85 µL/mL Tween 20 solution as a vehicle, were administered by oral gavage in a volume of 10 mL/kg. An additional group receiving only distilled water was excluded from the study to minimize animal usage, as previous research has demonstrated that Tween, saline, and water do not produce distinct behavioral effects (de Moraes Pultrini et al. 2006; Costa et al. 2011; Hazim et al. 2014).

#### 2.5.2 Experiment 1 Protocol

**FIGURE 1:**
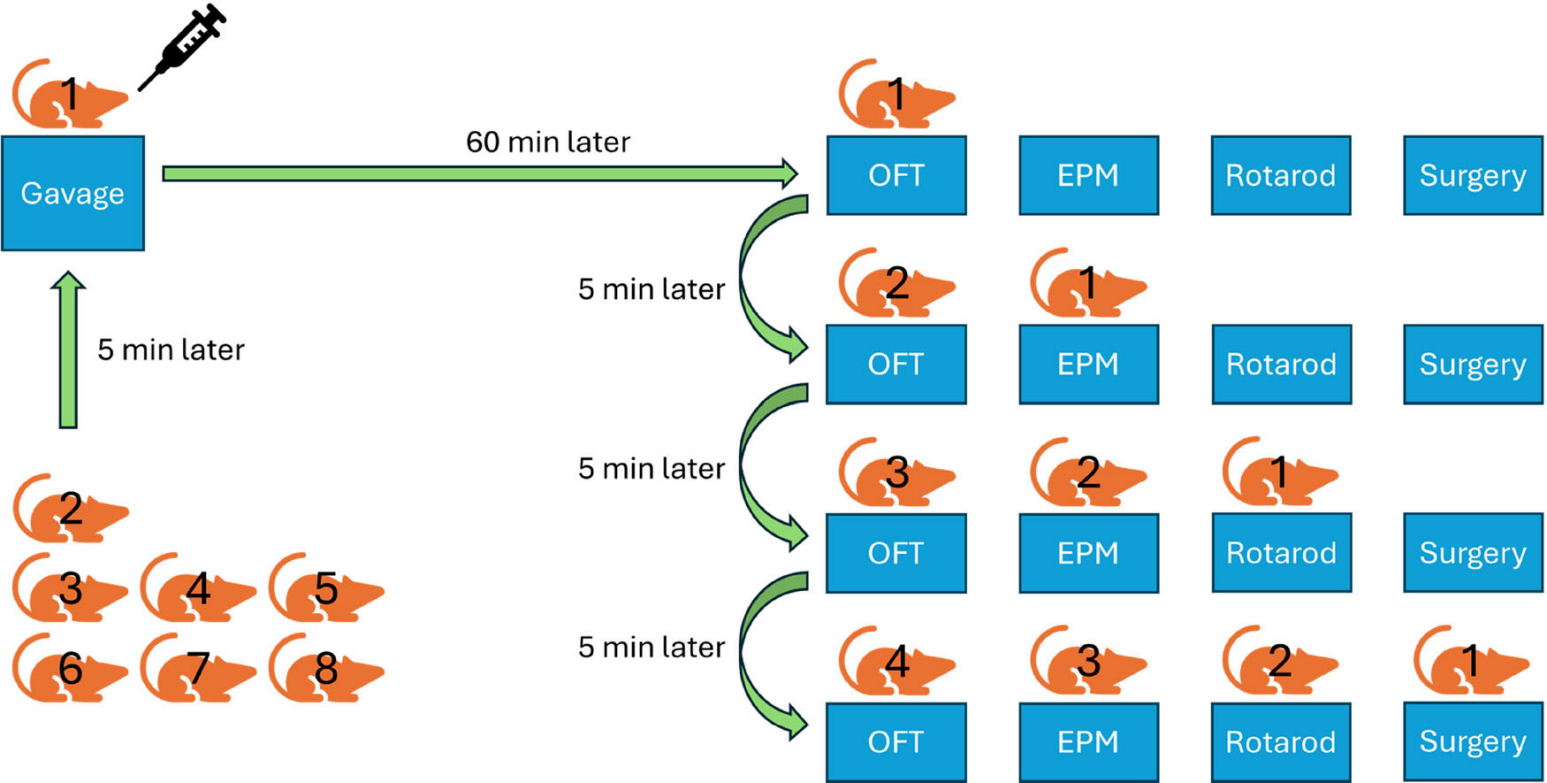
Timeline of Gavage administration and behavioral testing. OFT, and EPM.

Following a seven‐day adaptation, each day, a different group was tested. At 08:00, the rats were weighed, and solutions were freshly prepared accordingly. At approximately 09:00, the administration of oral gavage commenced, utilizing a 16‐gauge, curved‐tip, stainless steel cannula. Oral gavage was performed sequentially at five‐minute intervals for each animal, administering either the vehicle, diazepam (1.5 mg/kg), or *Salvia heldreichiana* essential oil (200 mg/kg), according to group assignment. This procedure ensured that the first behavioral test was conducted approximately 60 min after the gavage administration for all animals. The first rat underwent the OFT for five minutes. Afterward, the first rat was placed in the EPM test for five minutes, while the second rat was placed in the OFT. After five minutes of testing, the first rat was placed in the rotarod test, the second rat in the EPM test, and the third rat in the OFT. In this way, the rats changed stations for successive five‐minute periods (Figure 1). Thus, the effect of the administered solutions could be tested at similar time periods for each animal. After testing, the rat at the last station was anesthetized intraperitoneally with 90 mg/kg ketamine and 10 mg/kg xylazine. Intracardiac blood was collected, centrifuged at 5,000 rpm for 10 min, and stored at −80°C. The bilateral hippocampus was excised using the method described by Spijker (2011) and stored at −80°C.

#### 2.5.3 Experiment 2 (Repeated Dose)

In Experiment 2, 24 rats were divided into three groups of eight with similar weight: control, diazepam (1.5 mg/kg diazepam) and Salvia (200 mg/kg *Salvia heldreichiana* essential oil). All substances were administered via oral gavage in a volume of 10 ml/kg once daily for five days. A distilled water group was excluded from the study for the same reasons (de Moraes Pultrini et al. 2006; Costa et al. 2011; Hazim et al. 2014).

#### 2.5.4 Experiment 2 Protocol

Following a seven‐day adaptation period, oral gavage was performed once daily for five consecutive days between 09:00 and 10:00, administering either the vehicle, diazepam (1.5 mg/kg), or *Salvia heldreichiana* essential oil (200 mg/kg) according to group assignment. On the fifth day, behavioral tests occurred 60 min post‐gavage, following the same five‐minute interval procedure as shown in Figure 1. After testing, the rat was anesthetized, and tissue samples were collected as described in Experiment 1.

### 2.6 Measurement of Hippocampus and Serum Samples

Hippocampus tissues were weighed. Cold phosphate buffered saline (PBS‐Phosphate Buffered Saline, pH 7.4) was added to the tissue at a ratio of 1:9 (Tissue weight (g) : PBS (mL)) according to the manufacturer's instructions. Hippocampus tissues were homogenized using both a mechanical (PRO Scientific Inc., PRO 200) and ultrasonic homogenizer (OMNI International Inc., Omni‐Ruptor 4000). The homogenate was then centrifuged at 15,000 × *g* for 10 min at −4°C. The supernatant was used for total protein measurement (BCA, ABP Biosciences) and ELISA kits. Serotonin (Bioassay Technology Laboratory, E0866Ra), noradrenaline (Bioassay Technology Laboratory, EA0041Ra) and GABA (Bioassay Technology Laboratory, E0102Ra) were measured according to the manufacturer's instructions. Neurotransmitter levels were expressed as a ratio to total hippocampal protein (e.g., ng/mg, ng/g or nmol/g protein).

The ELISA method (Bioassay Technology Laboratory, E0828Ra), was used to measure serum cortisol level. Serum ALT, AST, creatinine, and BUN levels were measured by the colorimetric method using a biochemistry analyzer (Mindray, BS‐400).

### 2.7 Statistical Analysis

Statistical evaluation of the data was conducted using IBM SPSS 25. Within‐group comparisons were made for Experiment 1 and Experiment 2. Since the sample size was small, the differences between the groups were analyzed by the Kruskal–Wallis test. Dunn's test with Bonferroni correction was performed to determine the group(s) from which the difference originated in the variables in which there was a difference. The significance level of *p* < 0.05 was accepted, and data were presented as mean ± standard deviation in the text. Data distribution was depicted with boxplots when presenting continuous data.

## 3 Results

### 3.1 Behavioral Tests Results

**FIGURE 2:**
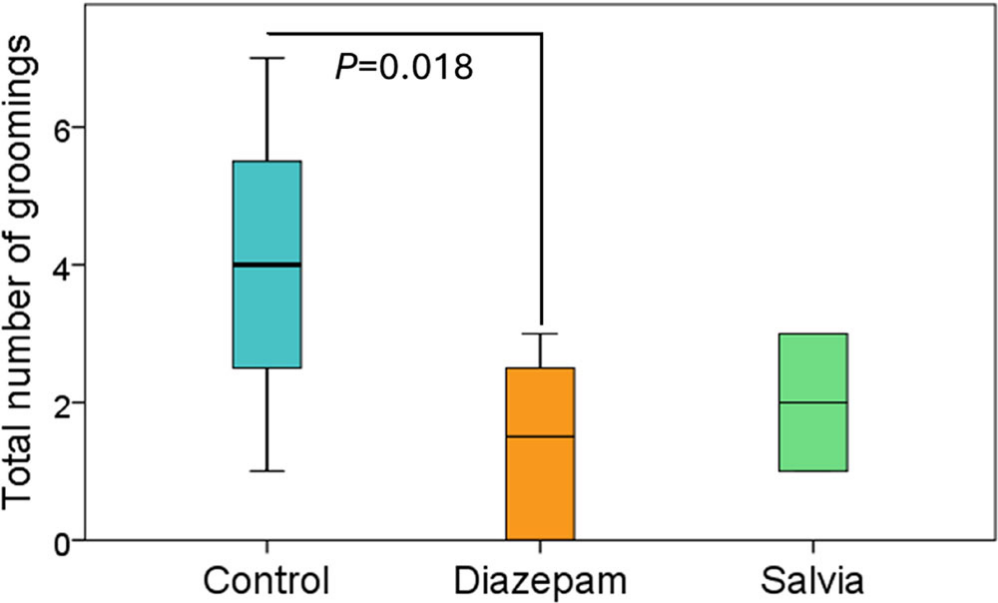
Experiment 1, Open field test and total number of groomings.

**FIGURE 3:**
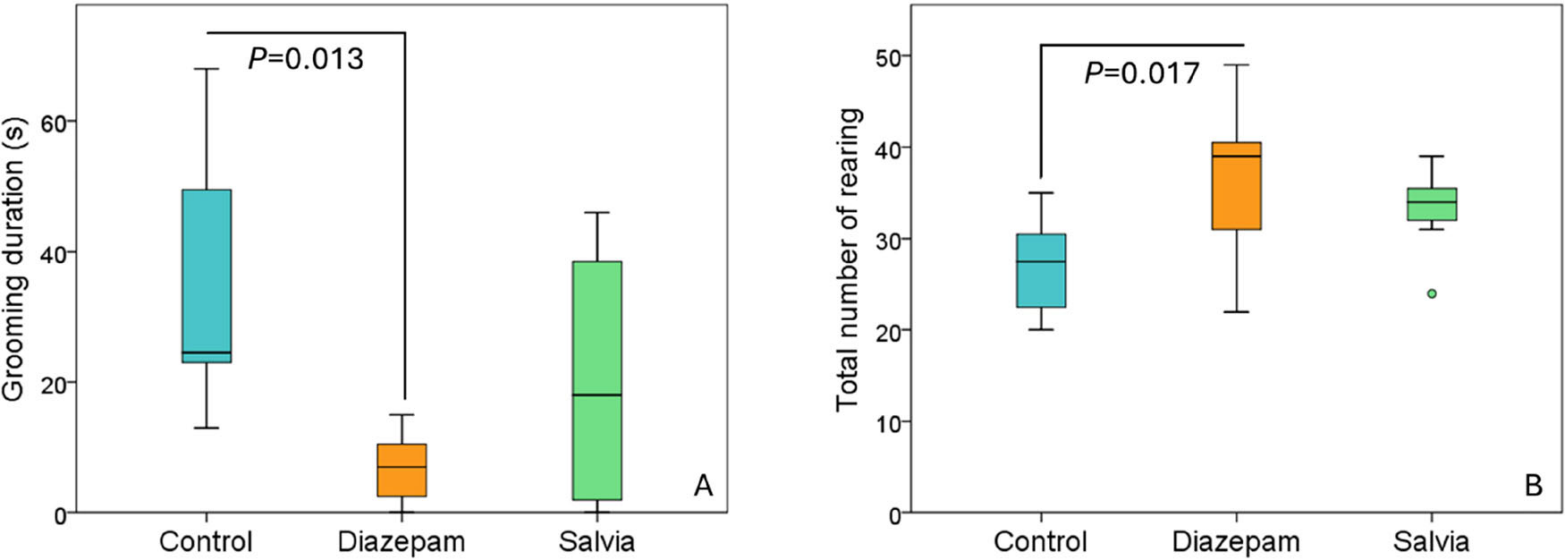
A Experiment 2: OFT and grooming duration B. Experiment 2; OFT and total number of rearing.

In Experiment 1, the total number of grooming in the OFT was lower in the diazepam group (1.38 ± 1.30) than in the control group (4.00 ± 2.00) (*p* = 0.018) (Figure 2). In the OFT in Experiment 2, the grooming duration was shorter in the diazepam group (6.88 ± 5.19 s) compared to the control group (34.38 ± 19.57 s) (*p* = 0.013) (Figure 3.A). In the OFT in Experiment 2, the total number of rearing was higher in the diazepam group (36.50 ± 9.02) compared to the control group (27.00 ± 5.13) (*p* = 0.017) (Figure 3.B). Although no statistically significant difference was observed, a similar trend was noted in the Salvia group. No significant difference was observed between the groups in both experiments in the EPM test and the rotarod test (data not shown). In the EPM test, four animals in Experiment 1 and five in Experiment 2 fell from the open arms of the apparatus, resulting in a total of nine animals whose data were not evaluated.

### 3.2 Results for Hippocampus Tissue

**FIGURE 4:**
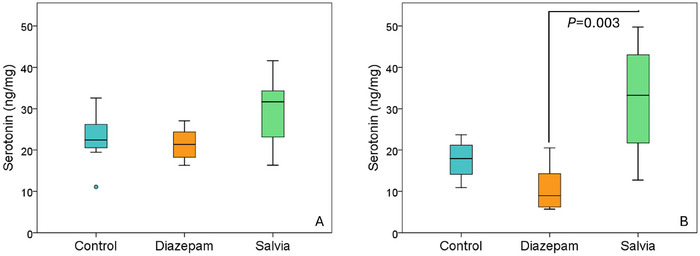
A Experiment 1: hippocampal serotonin levels. B Experiment 2: hippocampal serotonin levels.

In both experiments, there was no significant difference between the Salvia group and the control group regarding hippocampal serotonin, noradrenaline and GABA levels (Table 1 and Table 2). In Experiment 2, hippocampal serotonin levels in the Salvia group (32.31 ± 13.01 ng/mg) were significantly higher than in the diazepam group (10.77 ± 5.69 ng/mg) (*p* = 0.003). Similar trends, though not statistically significant, were observed in Experiment 1 (Figure 4A and 4B). In Experiment 1, hippocampal noradrenaline levels were lower in the Salvia group (62.08 ± 22.66 ng/g) than in the diazepam group (112.82 ± 35.91 ng/g) (*p* = 0.016). Additionally, in Experiment 1, hippocampal GABA levels were higher in the diazepam group (298.27 ± 63.86 nmol/g) compared to the control group (207.29 ± 24.13 nmol/g) (*p* = 0.014).

**TABLE 1:** Experiment 1: Hippocampal noradrenaline, GABA, and serotonin results.

			Experiment 1		
			Control	Diazepam	Salvia
			(*n* = 8)	(*n* = 8)	(*n* = 8)
Hippocampus	Noradrenaline (ng/g)	Median	61.42	126.61	57.01^b^
	(*R* ^2^ = 0985)	Mean	61.13	112.82	62.08
	GABA (nmol/g)	Median	199.66	319.65^a^	209.10
	(*R* ^2^ = 0996)	Mean	207.29	298.27	228.89
	Serotonin (ng/mg)	Median	22.40	21.32	31.68
	(*R* ^2^ = 0994)	Mean	22.75	21.40	29.52

*Note*: Different letters (e.g., a and b) denote statistically significant differences between groups. a: *p* < 0.05 vs control, b: *p* < 0.05 vs diazepam.

**TABLE 2:** Experiment 2: Hippocampal noradrenaline, GABA and serotonin levels.

			Experiment 2		
			Control	Diazepam	Salvia
			(*n* = 5)	(*n* = 3)	(*n* = 8)
Hippocampus	Noradrenaline (ng/g)	Median	74.23	39.37	127.43
	(*R* ^2^ = 0997)	Mean	79.31	39.70	117.90
			Control	Diazepam	Salvia
			(*n* = 8)	(*n* = 8)	(*n* = 8)
	GABA (nmol/g)	Median	207.95	227.54	250.70
	(*R* ^2^ = 0957)	Mean	219.83	227.96	245.70
			Control	Diazepam	Salvia
			(*n* = 7)	(*n* = 6)	(*n* = 8)
	Serotonin (ng/mg)	Median	17.93	8.96	33.26^b^
	(*R* ^2^ = 0994)	Mean	17.59	10.77	32.31

*Note*: Different letters (e.g., a and b) denote statistically significant differences between groups. a: *p* < 0.05 vs control, b: *p* < 0.05 vs diazepam.

### 3.3 Results of Serum Parameters

**FIGURE 5:**
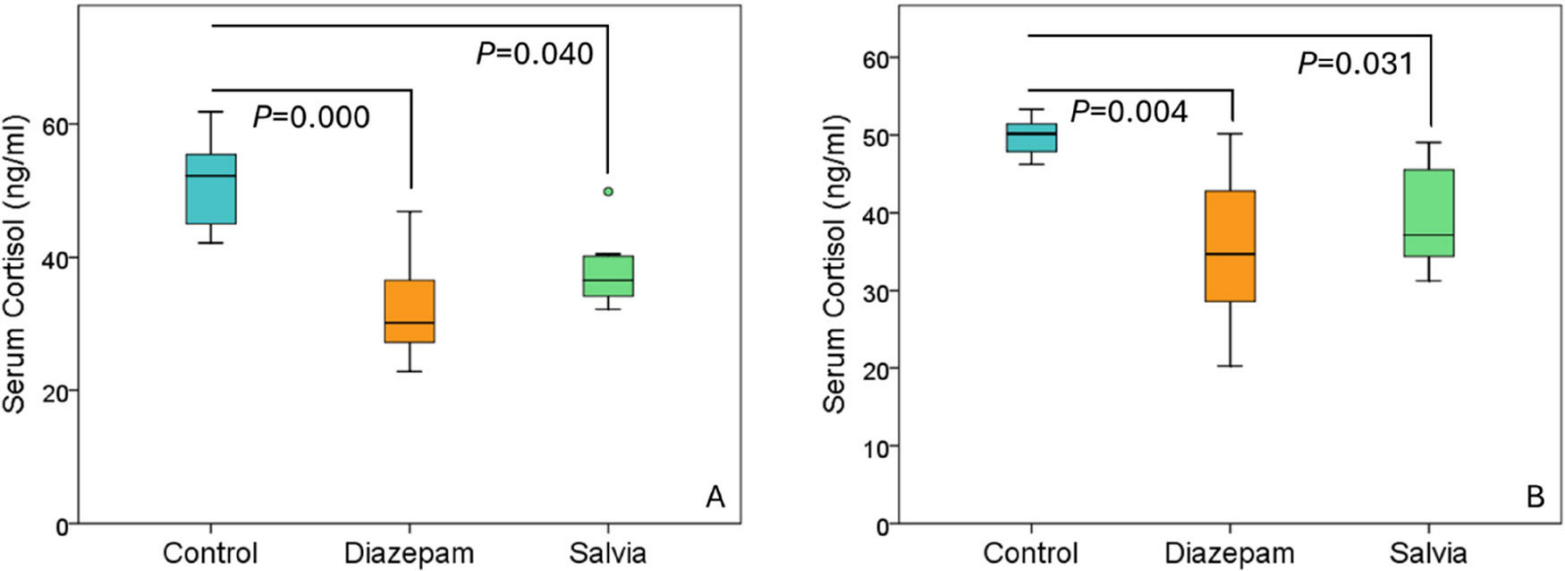
A Experiment 1: serum cortisol levels B. Experiment 2: serum cortisol levels.

In both experiments, serum cortisol levels were lower in Salvia and diazepam groups compared to the control group (Figure 5, Table 3) [Experiment 1: Control group (51.15 ± 6.84 ng/ml), diazepam group (32.17 ± 7.98 ng/mL), salvia group (37.97 ± 5.65 ng/mL), *p* < 0.05; Experiment 2: Control group (49.77 ± 2.60 ng/mL), diazepam group (35.33 ± 9.79 ng/mL), Salvia group (39.31 ± 6.62 ng/mL), *p* < 0.05; In Experiment 2, the extreme outlier of the seventh animal in the control group was removed]. ALT, AST, BUN and creatinine levels of the Salvia group were similar to the control group (data not shown). Creatinine levels were similar between the groups in Experiment 1, whereas creatinine levels in the diazepam group (0.51 ± 0.02 mg/dl) were lower than the control (0.54 ± 0.02 mg/dl) and Salvia groups (0.54 ± 0.02 mg/dl) in Experiment 2 (*p* < 0.05).

**TABLE 3:** Experiment 1 and 2, serum cortisol levels.

			Experiment 1		
			Control	Diazepam	Salvia
			(*n* = 8)	(*n* = 8)	(*n* = 8)
Serum	Cortisol (ng/mL)	Median	52.23	30.12^a^	36.54^a^
	(*R* ^2^ = 0999)	Mean	51.15	32.17	37.97
		Experiment 2			
		Control	Diazepam	Salvia	
		(*n* = 7)	(*n* = 8)	(*n* = 8)	
	Cortisol (ng/mL)	Median	50.17	34.70^a^	37.16^a^
	(*R* ^2^ = 0999)	Mean	49.77	35.33	39.31

*Note: Different letters (e.g., a and b) denote statistically significant differences between groups. a: p < 0.05 vs control, b: p < 0.05 vs diazepam*.

## 4 Discussion

Scientific studies conducted to understand the physiological and pathophysiological characteristics of anxiety may contribute to the development of new options for the treatment of anxiety disorders by increasing knowledge in this field. In particular, there is a need for an ideal anxiolytic for the acute treatment of anxiety disorders that is fast‐acting and safe in terms of side effects (Stein et al. 2010). While studies for this elusive goal continue, patients who cannot access treatment, cannot continue treatment due to side effects, or do not benefit from treatment turn to complementary treatment options. Among complementary therapies used for anxiety, the use of herbal medicines ranks first. *Passiflora incarnata*, *Valeriana officinalis*, and *Piper methysticum* are frequently preferred for this purpose (Ernst 2006; Lakhan and Vieira 2010). In the literature, many plants including *Salvia* species, have been reported to show anxiolytic effects. However, no other study investigating the anxiolytic effect of *Salvia heldreichiana*, which is endemic for Türkiye, has been found; to the best of our knowledge, our study is the first study on this subject.

The most important finding of the study is that *Salvia heldreichiana* essential oil decreased serum cortisol levels in both experiments. This effect was also observed in the diazepam group, with a known anxiolytic effect. Many types of psychological stress cause an increase in cortisol synthesis and release. There are studies reporting high blood cortisol levels in anxiety disorders (Feder et al. 2017). While the main glucocorticoid in humans is cortisol, the main glucocorticoid in rats is corticosterone. However, cortisol is also produced in rats in stressful situations (Ayada et al. 2002). Gong et al. showed that corticosterone and cortisol are correlated and that cortisol increases faster than corticosterone during acute stress (Gong et al. 2015). Cortisol levels were observed to be high in the blood of rats under stress (Yin et al. 2007, Bhat et al. 2007). In contrast, Azarfarin et al. showed that diazepam decreased serum cortisol levels in rats (Azarfarin et al. 2018). The fact that *Salvia heldreichiana* essential oil decreased serum cortisol levels similar to diazepam indicates that the essential oil may have anxiolytic effects. In addition, *Salvia heldreichiana* essential oil can also be tested in new studies modeling diseases in which cortisol elevation is observed in pathophysiology.

In behavioral tests, the diazepam group (positive control) exhibited an anxiolytic‐like effect only in the OFT and only in the supportive parameters (Roth and Katz 1979) (increase in the total number of rearing, decrease in the number and duration of grooming). The Salvia group showed similar trends, but these results were not statistically significant. Increasing the sample size or dose of *Salvia heldreichiana* essential oil may yield statistically significant findings. It is noteworthy that the anxiolytic‐like effects of the diazepam group were not observed in the main parameters (number of entries to the center, time spent in the center, number of thigmotaxis, duration of thigmotaxis, and number of defecations). In addition, there was no finding that diazepam decreased anxiety‐like behaviors in the EPM test. This lack of anxiolytic response could be attributed to an insufficient dose of diazepam or an extended interval before testing. However, Liu et al. reported that the same dose of diazepam reduced anxiety‐like behaviors in rats 60 min post‐administration (Liu et al. 2015). In order to interpret these contradictory results in a consistent manner, it is essential to consider that unconditioned anxiety tests may inadequately provoke conflict (Ennaceur 2014) and may not always give consistent results with each other (Radhakrishnan and Gulia 2018). In the Rotarod test, since no differences were observed between the groups in both experiments, 1.5 mg/kg p.o. diazepam and 200 mg/kg *Salvia heldreichiana* essential oil were not considered to have a sedative effect.

The similarity between rodents and humans is greater in subcortical than in cortical structures. The hippocampus, a subcortical structure, was the focus of this study due to its role in anxiety physiology, its connections with the prefrontal cortex and amygdala, and its involvement in processing contextual information. There are also other studies in the literature investigating the levels of hippocampal serotonin, GABA, and noradrenaline following unconditioned anxiety tests (Wu et al. 2020; Feng et al. 2018; Zhang et al. 2019). In both experiments, hippocampal serotonin levels were higher in the Salvia group than in the diazepam group, reaching statistical significance in Experiment 2. Although not statistically significant, serotonin levels were also higher in the Salvia group compared to the control group in both experiments. This difference became more pronounced in Experiment 2 (Figures 4A and 4B). Based on these results, it can be predicted that prolonging the administration duration may result in significant differences between the Salvia and control groups. The lack of differences in hippocampal noradrenaline and GABA levels between the Salvia and control groups suggests that the potential anxiolytic effect of *Salvia heldreichiana* essential oil may be mediated through cortisol and/or serotonin pathways. In Experiment 1, the hippocampal noradrenaline levels in the diazepam group were found to be higher than those in the Salvia group. However, few studies in the literature have investigated the effect of diazepam on hippocampal noradrenaline levels. Contrary to our findings, Broderick (Broderick 1997) reported that diazepam decreases hippocampal noradrenaline release. Another interesting finding from Experiment 1 is that hippocampal GABA levels in the diazepam group were significantly higher compared to those in the control group. It is important to note that diazepam exerts its effects by modulating GABA‐A receptors rather than directly increasing GABA levels (Kas and Olivier 2020). In support of this, Carton et al. (Carton et al. 2022) also observed no change in hippocampal GABA levels following acute administration of diazepam. The effect of diazepam on hippocampal noradrenaline and GABA was not observed in Experiment 2. Further research is required to investigate how diazepam impacts noradrenaline and GABA levels in the hippocampus.

Finally, ALT, AST, creatinine, and BUN levels, assessed for potential hepatotoxic and nephrotoxic effects of *Salvia heldreichiana* essential oil, did not increase in either experiment. These results indicate that oral administration of *Salvia heldreichiana* essential oil at a dose of 200 mg/kg/day does not exhibit hepatotoxic or nephrotoxic effects.

In the study, the anxiolytic effects of *Salvia heldreichiana* essential oil were examined, and it was observed that this oil decreased serum cortisol levels like diazepam. This finding suggests that *Salvia heldreichiana* essential oil may have an anxiolytic effect. Additionally, the oil showed a tendency to increase hippocampal serotonin levels, further supporting its anxiolytic potential. In behavioral tests, a trend was observed that *Salvia heldreichiana* essential oil reduced anxiety‐like behaviors in some parameters only in the OFT. Similarly, diazepam, which is known to have anxiolytic effect, showed a significant effect only on a few parameters only in the OFT. When both the anxiolytic‐like trends observed in behavioral tests and the variations in serum cortisol and hippocampal serotonin levels are evaluated together, it can be posited that *Salvia heldreichiana* exhibits potential for anxiolytic effects. There is a need for further investigation into the potential anxiolytic effects of *Salvia heldreichiana* essential oil, including conditional anxiety tests and varying doses and administration periods.

## Author Contributions

**Ahmet Onur DASTAN**: Conceptualization, animal experiments, biochemical analyses, writing. **Asli San DAGLI GÜL**: Animal experiments, biochemical analyses, writing. **Gülderen YILMAZ**: Collecting plant material. **Okan ARIHAN**: Animal experiments, biochemical analyses, writing. **Aysen ERDEM**: Writing.

## Conflicts of Interest

The authors declare no conflicts of interest.

## Peer Review

The peer review history for this article is available at https://publons.com/publon/10.1002/brb3.70701

## Data Availability

The data that support the findings of this study are not available.
